# Removal of Heavy Metal Ions with Acid Activated Carbons Derived from Oil Palm and Coconut Shells

**DOI:** 10.3390/ma7053634

**Published:** 2014-05-07

**Authors:** Mokhlesur M. Rahman, Mohd Adil, Alias M. Yusof, Yunus B. Kamaruzzaman, Rezaul H. Ansary

**Affiliations:** 1 Department of Pharmaceutical Chemistry, Faculty of Pharmacy, International Islamic University Malaysia, Kuantan 25200, Pahang, Malaysia; E-Mail: ansarychem@gmail.com; 2 Department of Biotechnology, Faculty of Science, International Islamic University Malaysia, Kuantan 25200, Pahang, Malaysia; E-Mail: kama@iium.edu.my; 3 Department of Chemistry, Faculty of Science, Universiti Teknologi Malaysia, UTM Johor Bahru 81310, Johor, Malaysia; E-Mails: m_adil@yahoo.com (M.A.); alias@kimia.fs.utm.my (A.M.Y.)

**Keywords:** adsorption, oil palm shell, coconut shell, activated carbon, heavy metal ions

## Abstract

In this work, batch adsorption experiments were carried out to investigate the suitability of prepared acid activated carbons in removing heavy metal ions such as nickel(II), lead(II) and chromium(VI). Acid activated carbons were obtained from oil palm and coconut shells using phosphoric acid under similar activation process while the differences lie either in impregnation condition or in both pretreatment and impregnation conditions. Prepared activated carbons were modified by dispersing hydrated iron oxide. The adsorption equilibrium data for nickel(II) and lead(II) were obtained from adsorption by the prepared and commercial activated carbons. Langmuir and Freundlich models fit the data well. Prepared activated carbons showed higher adsorption capacity for nickel(II) and lead(II). The removal of chromium(VI) was studied by the prepared acid activated, modified and commercial activated carbons at different pH. The isotherms studies reveal that the prepared activated carbon performs better in low concentration region while the commercial ones in the high concentration region. Thus, a complete adsorption is expected in low concentration by the prepared activated carbon. The kinetics data for Ni(II), Pb(II) and Cr(VI) by the best selected activated carbon fitted very well to the pseudo-second-order kinetic model.

## 1. Introduction

The ecological crisis of environmental pollution has been blamed on different issues, of which pollution due to metals or their species in the environment is the major one. Heavy metal pollution affects flora, fauna and other abiotic components of the ecosystem. As these toxins build up in our bodies, they block the receptor sites for essential minerals, so that minerals such as iron, calcium and magnesium cannot be utilized and absorbed to serve body and enzymes functionalities causing enzyme dysfunction, nutritional deficiencies, hormonal imbalances, neurological disorders, damages brain chemistry, and can even lead to auto-immune disorders, cancer, and other debilitating chronic conditions. For short- and long-term toxic effects, the maximum permissible concentrations of heavy metals in drinking water as well as in municipal and industrial discharges are closely regulated in most countries through proper legislation. As, ultimately, heavy metal regulations for both drinking water and wastewater are becoming stricter, the conventional means of water treatment becomes either costly or inefficient. It has been shown by many researchers [1–3] that lignocellulosic agricultural wastes, particularly nutshells, are very good candidates as precursors for the removal of heavy metal ions from aqueous solutions. Huge amounts of oil palm and coconut shells are produced as agricultural wastes in Malaysia. The utilization of these low cost agricultural wastes as carbon precursor is very promising [4–6], but their use as metal ion scavenger is rare. In this study, activated carbons were prepared from oil palm and coconut shells, using low temperature activation with phosphoric acid, suitable for removing heavy metal ions. A hydrated iron-oxide dispersed activated carbon was prepared from subsequent modification of acid activated carbon. The removal of divalent cations of nickel and lead, and the oxyanions of hexavalent chromium was tested by the developed adsorbents. The adsorption equilibrium and kinetics data are fitted to various models to evaluate and compare the performance with a commercial activated carbon.

## 2. Results and Discussion

### 2.1. Phosphoric Acid Activation Enhances Positive Ions/Elements Removal (Quantification of Surface Functional Groups by Boehm’s Titration)

Base neutralization capacity and acidic groups distributed on surfaces of prepared activated carbons, PSW-P-500 and PSW-P-ad-500 and on commercial activated carbon, Commercial Activated Carbon (CAC) were determined and quantified by Boehm’s titration and the results are tabulated in Table 1. In the literature, strong acidic groups detected by NaHCO_3_ are assumed to be only the carboxylic group. However, activated carbon prepared using H_3_PO_4_ might contain strongly acidic hydroxyl (–OH) groups due to the presence of phosphorous containing acids attached to the surface [3]. Thus, the consumption of NaHCO_3_ by the H_3_PO_4_ activated carbon quantified the carboxylic as well as the phosphorous acids as strong acidic groups. Sodium carbonate, in addition to strong acidic groups (Group I in Table 1), is capable of detecting lactones. Other than lactones, phosphorous containing acids of intermediate strength attached on carbon surface may also take part in the neutralization reaction with Na_2_CO_3_. Sodium hydroxide, in addition to the previously described groups, can detect phenols and phosphonic acids whereas sodium ethoxide (NaOEt), in addition to the all other described groups, can detect carbonyl groups.

The CAC can up take only very little NaOH, which indicates its lesser acidic nature due to phenolic and ketonic groups in quinones which is consistent with FTIR. The results of Boehm’s titration indicated that the total acidity of oil palm shell derived activated carbon (4.5–4.8 meq·g^−1^) is much higher than that of CAC (0.5 meq·g^−1^).

### 2.2. Determination of pH_ZPC_ of Activated Carbon (pH Drift Method)

**Figure 1.**
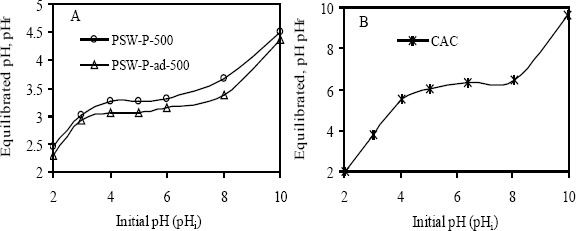
The pH_ZPC_ determination curve from the crossover point of equilibrated and initial pH by (**A**) produced activated carbon and (**B**) commercial activated carbons.

Experimental results of pH_ZPC_ determination, using pH drift method, are shown in Figure 1. Here, equilibrated pH (pH_f_) by activated carbon was plotted against initial pH (pH_i_) of the solution having constant ionic strength (0.01 M NaCl). A pH at a point where the initial pH of the solution crossover the final pH equilibrated by a certain amount of an adsorbent is referred to as the pH_ZPC_. The much lower pH_ZPC_ of prepared activated carbons indicates their very acidic nature (see Table 2). Since the pH_ZPC_ of CAC is 6.4, it contains no strong acidic group, which is consistent with the Boehm’s titration.

The effect of pH were tested to evaluate the feasibility and mechanism of adsorption. Since the basal structural units or graphitic layers of activated carbon are considered softer than the surface functional groups [7], the competitive effect of soft ion on the adsorption of borderline metal ion is very interesting. Adsorption of Cu(II), Ni(II) as borderline [8]; IUPAC, 2002 [9] and Pb(II) as soft ion IUPAC, 2002 [9] was conducted from a multi-solute solution to determine the selectivity order.

The value of pH_zpc_ was determined by pH-metric following that of Periasam *et al.* [10] and potentiometric titration, after Parfitt *et al.* [11] and Tewari [12]. Both the methods provide accurate values of pH_zpc_. It has been reported that the pH of carbon ranging between 4 and 8 is acceptable for most of the applications [13]. It was further established that the generated active carbon exhibited characteristics of high grade available carbon materials. This value of pH_zpc_ of between 3.1 and 6.4 meq·g^−1^, suggests that the surface release H^+^ ion into the bulk only when the pH of the solution is above 5.5 ± 0.1 meq·g^−1^ and the surface becomes positive when the solution pH is below 5.0 ± 0.1 meq·g^−1^. It thus indicates that below the pH of 5.0 ± 0.1 meq·g^−1^, water donates more protons than the hydroxide groups, and so the adsorbent surface is positively charged (attracting anions). Conversely, in the event of pH being higher than 5.0 ± 0.1 meq·g^−1^, the surface would be negatively charged (attracting cations/repelling anions). The more the pH of the solution rises above 5.0 ± 0.1 meq·g^−1^, the higher the electrostatic attraction between the surface and adsorbate of Pb(II) and Ni(II) ions. It is clear that in the case of granular carbon (considering a given bed of particles), activation with H_3_PO_4_ produces higher proportions of micro- and mesoporosity but a lesser proportion of macro-porosity.

### 2.3. Adsorption of Ni(II) and Pb(II)

**Figure 2.**
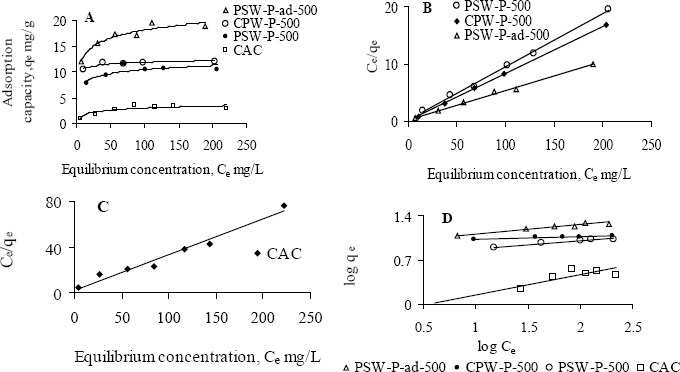
(**A**,**B**,**C**) Langmuir and (**D**) Freundlich adsorption isotherms of Ni(II) at initial pH 5 by various activated carbons while initial concentrations were varied, 6–227 mg·L^−1^.

**Figure 3.**
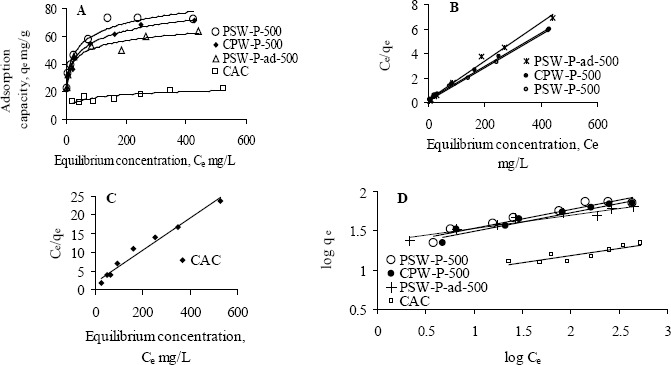
(**A**,**B**,**C**) Langmuir and (**D**) Freundlich adsorption isotherms of Pb(II) at initial pH 5 by various activated carbons while initial concentrations were varied, <50 to <600 mg·L^−1^.

Figures 2 and 3 show the Langmuir and Freundlich adsorption isotherms of Ni(II) and Pb(II), for an initial pH 5, by various activated carbons. The physical parameters for the adsorption of Ni(II) and Pb(II) are determined from the linear Langmuir (Figures 2B,C and 3B,C) and Freundlich (Figures 2D and 3D) isotherms and are tabulated in Table 3. One of the prepared activated carbons, PSW-P-ad-500 (derived from palm shells’ semi-dried impregnated feedstock), shows significantly higher Ni(II) adsorption capacity (Figure 2A,D). All prepared activated carbons have higher affinity towards Ni(II) as evident from the H-type class of the non-linear Langmuir isotherms (Figure 2A) according to the classification by Giles *et al.* [14]. The H-type class also reveals complete adsorption in low concentration. The higher affinity of prepared activated carbons towards Ni(II) implies that phosphoric acid activation enhances metal ion removal capacity.

The values of correlation coefficients of all Langmuir isotherms indicate that this model fits all the adsorption equilibrium data very well throughout the experimental range of study. Therefore, it is assumed that adsorption involves a direct contact of metal ions onto the surface of acid activated carbon and thus proceeds up to monolayer coverage. The performance of adsorbents for the removal of Ni(II) in aqueous solution are attributed from the maximum monolayer adsorption capacity, *q*_max_ and given as CAC << PSW-P-500 < CPW-P-500 << PSW-P-ad-500. All prepared activated carbons show higher adsorption capacity as well as stronger affinity towards Pb(II) which is also evident from the H-type isotherms (Figure 2A).

The commercial activated carbon has comparatively lower affinity towards Pb(II). A comparison in the removal capacity of Pb(II) by prepared and commercial activated carbons using the observed *q*_max_ determines the order of adsorbents according to their performance, under the experimental range of study, as CAC< PSW-P-ad-500< CPW-P-500 ≤ PSW-P-500. The adsorption data by all activated carbons also fitted well to the Freundlich model, but the best fitting is observed with the Langmuir model. The very low affinity of commercial activated carbon towards Ni(II) is also recognized from the significantly lower values of Freundlich constants, *K*_F_ and “*n*”. The highest adsorption capacity (*q*_max_) of Pb(II) is observed by one of the activated carbons prepared from “wet-feedstock”. Since two of the activated carbons, one from palm shell, PSW-P-500 and another from coconut shell, CPW-P-500, were obtained from the wet-feedstock, they have identical adsorption capacity, *q*_max_ for Pb(II). The comparatively higher values of *K*_F_ and “*n*” indicate that Pb(II) is more strongly bound onto the surface of PSW-P-ad-500. The effectiveness of adsorbents to be used in drinking water purification depends upon its complete removal efficiency of heavy metals ions at low concentration. Thus the model parameters (*q*_max_, *K*_F_ and *n*) indicate that the prepared activated carbon PSW-P-ad-500 is the best in this experiment in removing heavy metal ions such as Ni(II) and Pb(II) in the low concentration range. The equilibrium concentrations and fractions in the removal of Ni(II) and Pb(II), in the low concentration, are shown in Table 4 to verify the suitability of acid activated carbons.

**Figure 4.**
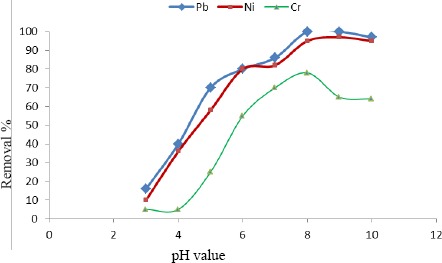
Comparison between % removal of Multiple Components (Pb, Ni and Cr) and final pH for 1.0 g·L^−1^ dose of PSW-P-ad-500.

Table 5 and Figure 4 show that the uptake capacity of multiple components followed the trend of Pb ≥ Ni >> Cr, which is consistent with the ascending electronegativity of these metals which are 1.85, 1.854, and 1.60 respectively (*from the periodic table according to Pauling*). Since the activated carbon is negatively charged, the potential of the electrostatic adsorption among the three adsorbates increases directly proportional to their electronegativity.

Based on batch equilibrium studies, the uptake capacity of the three heavy metals studied appears to be greatest for Pb, followed by Ni and then Cr. The presence of multiple heavy metals in the solution promoted a competition amongst them during the adsorption process. By checking the uptake of the three heavy metals in the single component experiments on a molar basis, the greatest removal achieved was for Pb followed closely by Ni and then Cr. In the multi-component experiment for the same carbon dose of 1.0 g·L^−1^, one can fnd that the uptake capacity was greater for Pb than Ni followed by Cr. To conclude, Cr was significantly affected by the presence of other heavy metals. Ni was affected to a lesser degree. Amazingly, the removal of Pb has improved as shown in Table 5.

### 2.4. Adsorption of Cr(VI)

#### 2.4.1. The Effect of pH

**Figure 5.**
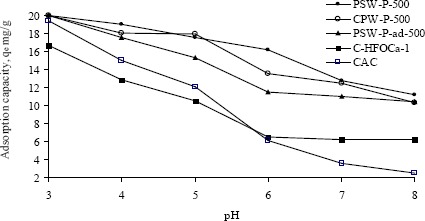
Adsorption capacity of Cr(VI) by various activated and modified carbon while pH were varied from 3 to 8 keeping initial concentration constant at ~40 mg·L−1.

The pH of solution plays an important role in the adsorption of cations and anions as described in the surface complex formation model (SCF) [15]. According to this model, the surface functional groups of activated carbon can be modeled as a single, weak diprotic acid and can be represented by the following surface reactions:

$${\text{SOH}}_{2}^{+}↔\text{SOH}+H_{S}^{+}$$ (1)

$$\text{SOH}↔{\text{SO}}^{-}+H_{S}^{+}$$ (2)

where, the symbol (SO^−^) represents the active site of the surface and H_s_^+^ is the activity of the proton at the solid surface. Therefore, an increase in solution pH releases protons from the surface thus exposing more negative sites (SO^−^) to bind more cations. In contrast, a decrease in pH protonates the surface exposing more positively charged sites on carbon to bind more anions. The effect of pH on the adsorption of oxyanions of Cr(VI) onto the various adsorbents was studied at varying pH from 3 to 8 while initial concentrations were kept constant at ~40 mg·L^−1^ and the results are shown in terms of adsorption capacity against pH in Figure 5. All adsorbents, except modified ones show identical adsorption capacity at pH 3 while the capacity decreases with the increase of pH. The adsorption capacities of three acid activated carbons remain almost similar over the range of pH 3 to 5. The adsorption capacity of commercial activated carbon was greatly decreased in the pH range from 6 to 8. Although the modification of acid activated carbon was made to bind more Cr(VI) on the protonated surface of hydrated iron oxide, its capacity is found to be lower. All prepared adsorbents have significantly higher adsorption capacity than that of commercial ones over the pH range of 5–8. From Figure 3 the value of pH_zpc_ suggests that the surface release of H^+^ ion into the bulk occurs only when the pH of the solution is above 5.5 ± 0.1 and the surface becomes positive when the solution pH is below 5.0. It thus indicates that below the pH 5.0, water donates more protons than the hydroxide groups, and so the adsorbent surface is positively charged (attracting anions). The adsorption capacities of one of the activated carbons, PSW-P-ad-500 and modified adsorbent, C-HFOCa-1, remain stable with the change of pH from 6 to 8 which is resulted probably from stronger bond strength.

### 2.5. Equilibrium Studies

**Figure 6.**
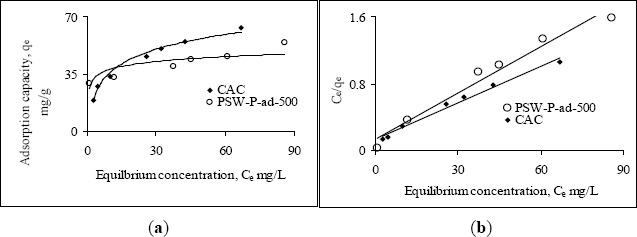
Langmuir adsorption isotherms of Cr(VI) by activated carbons, CAC and PSW-P-ad-500, at initial pH 3 while initial concentrations were varied from 40 to <600 mg·L−1. (a) Nonlinear Langmuir adsorption isotherms; (b) Linear Langmuir adsorption isotherms.

**Figure 7.**
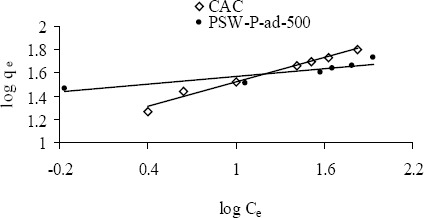
Freundlich adsorption isotherms of Cr(VI) by activated carbons, CAC and PSW-P-ad-500, at initial pH 3 while initial concentrations were varied from 40 to <600 mg·L−1.

The adsorption behavior of one of the acid activated carbons, PSW-P-ad-500 for Cr(VI) was compared with commercial ones by fitting the adsorption equilibrium data to Langmuir and Freundlich models. Figure 6a,b shows the non-linear and linear Langmuir adsorption isotherms while Figure 7 the Freundlich isotherms. The isotherms reveal that the adsorption capacity of acid activated carbon is higher in the low concentration region while that of commercial activated carbon is higher in the high concentration region. The model parameters are presented in Table 6. All the data fitted very well in both the Langmuir and Freundlich models. The higher adsorption capacity in the low concentration region indicates stronger bond strength to bind Cr(VI) as well as comparatively higher suitability of the adsorbents to be used in drinking water purification purposes. The equilibrium concentrations and fractions in the removal of Cr(VI) in the low concentration range are shown in Table 7 to show the suitability of acid activated carbon over commercial ones.

### 2.6. Adsorption Kinetics

**Figure 8.**
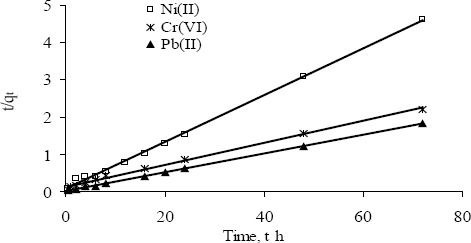
A linear graph of pseudo second-order kinetic model for the adsorption of Ni(II), Pb(II) and Cr(VI) while their initial concentrations were kept constant at 43 ± 2, 95.2 ± 0.5 and 77.4 ± 0.8 mg·L−1 respectively and contact time was varied from 0.5 to 72 h.

The adsorption kinetics data of Ni(II), Pb(II) and Cr(VI) by PSW-P-ad-500 fitted well to the Freundlich and the Langmuir models (Figure 8). The kinetic model parameters such as rate constant, *k*_2ad_ and calculated adsorption equilibrium capacity, *q*_e_ are determined and presented in Table 8. The first-order reaction model was used to check all the results but the correlation coefficient is not high. However, the rate law for a pseudo-second order could be fixed with very high correlation coefficient. The sorption of metal ion onto adsorbent could be a pseudo-second order process rather than first-order.

The higher values of corresponding correlation coefficients indicate that the Freundlich and the Langmuir models fitted all the kinetics data very well.

## 3. Experimental

### 3.1. Reagents and Chemicals

The reagents used to pretreat and impregnate the raw materials were sulphuric acid (98%) purchased from Merck (Darmstadt, Germany) and phosphoric acid (85%) from Mallinckrodt (Utrecht, The Netherlands). The chemicals and reagents used for preparing metal ion solutions, modifying carbon and detecting Cr(VI) were Ni(II) nitrate hexahydrate (Fluka), lead (II) nitrate (Riedel-deHaën), and potassium dichromate (Merck ), nitric acid (65%, Merck), hydrochloric acid (37%, Mallinckrodt), sodium hydroxide pellets (Merck), iron (III) chloride-6-hydrate (Hamburg Chemicals), calcium (II) hydroxide (GCE), sodium chloride (Merck), 1,5-diphenylcarbazide (Merck) and acetone. All chemicals and reagents were of analytical grade.

### 3.2. Activated Carbon Preparation

#### 3.2.1. Raw Material

Commercial activated carbon, “Aktivkohle”, (abbreviated as, CAC) was obtained from Riedel-deHaën, Germany. Oil palm kernel shells (P) were collected from local palm-oil processing factory and then repeatedly washed with tap water followed by drying in the sunlight. The dried palm kernel shells were crushed using a mechanical grinding machine and sieved to the selected particle sizes, 1.18–2.36 mm. The selected crushed particles were immersed in aqueous solution of 30% sulphuric acid (-SW-) for 24 h in a bucket and then washed and dried. This step is doped as pretreatment.

#### 3.2.2. Activated Carbon

Prior to activation, prepared raw materials were impregnated with 42.5% aqueous solution of H_3_PO_4_ (-P-), in a wt% ratio of 1:1 (against H_3_PO_4_ on the basis of 100% purity), under different impregnation conditions. The physical state of impregnated feedstock, which was either wet by the impregnation solution or in the form of a certain degree of dryness (e.g., apparently/semi-dried [-ad-], by the evaporation, before charging into the furnace) was termed as charge state. Activation was carried out in a porcelain casserole placed in a Carbolite muffle furnace. Residence time at activation temperature was maintained at one hour. After activation, the products were thoroughly washed in a soxhlet’s apparatus with distilled water to about neutrality. The removal of all adhered phosphates from the granulated product was confirmed by adding a few drops of 30% solution of Pb(NO_3_)_2_ to the rinsed water. Finally the product was dried in an oven at 110 °C overnight and stored for subsequent characterization and adsorption studies. The preparation variables are shown in Table 9.

Based on Guo and Lua [16] who studied the effect of pretreatment on oil-palm stone, the acidic groups were shown to be well developed from the samples pretreated with 5%–30% H_2_SO_4_. A 30% solution of H_2_SO_4_ was used in the pretreatment step to enhance surface acidity. To maximize development of internal surface area as well as to enhance development of various acidic surface groups, pretreatment was also explored with 30% H_3_PO_4_. Some literatures showed that activated carbon prepared from lignocellulosic precursor with H_3_PO_4_ and air has very high metal ion adsorption capacity owing to the presence of oxygen and phosphorus bearing acidic groups [1–3].

The effects of structural properties and their changes during cellulose hydrolysis on the enzymatic hydrolysis rate have been studied from the reaction mechanism point of view. Important findings are listed as follows: (1) The crystallinity index of partially crystalline cellulose increases as the hydrolysis reaction proceeds, and a significant slowing down of the reaction rate during the enzymatic hydrolysis is, in large part, attributable to this structural change of cellulose substrate; (2) The crystallinity of completely disordered cellulose, like phosphoric-acid-treated cellulose, does not change significantly, and a relatively high hydrolysis rate is maintained during hydrolysis; (3) The specific surface area (SSA) of partially crystalline cellulose decreases significantly during enzymatic hydrolysis while the change in SSA of regenerated cellulose is found to be negligible; (4) The value of degree of polymerization (DP) of highly ordered crystalline cellulose remains practically constant whereas the change in DP of disordered regenerated cellulose is found to be very significant; (5) Combination of these structural effects as well as cellulose adsorption, product inhibition, and cellulase deactivation all have important influence on the rate of cellulase reaction during cellulose hydrolysis. More experimental evidence for a two-phase model, which is based on degradation of cellulose by simultaneous actions of cellulase complex on the crystalline and amorphous phases, has been obtained. Based on experimental results from this study and other results accumulated, the mode of cellulose action and a possible reaction mechanism are proposed.

Three activated carbons were prepared from oil palm and coconut shells. The shells were collected, washed, dried, crushed and sieved to the particle sizes of 1.18–2.36 mm. Selected particles were subjected to acid wash, prior to impregnation with 42% aqueous H_3_PO_4_ in a weight ratio 1:1, which is called pretreatment. The physical state of impregnated feedstock, which was either wet by the solution or was semi-dried by the evaporation of solution, before charging into the furnace, was termed as charge state. The carbonization was carried out at 500 °C with 1 h hold time in a porcelain casserole placed in the muffle furnace. The chamber of the Carbolite muffle furnace is not air tight according to the product specification (Model: ELF 11/6B, Barloworld Scientific, Stone, Staffordshire, UK). To enhance metal ion uptake capacity by concurrent activation/oxidation, acid pretreated precursors were impregnated with phosphoric acid and carbonized in the Carbolite muffle furnace. Iron content in the carbon-HFO composite was determined using energy dispersive X-ray analysis (EDAX) and found an average of 7.5% as Fe. The products were thoroughly washed to about neutrality in a Soxhlet’s apparatus, then dried in an oven at 110 °C and stored. The preparation variables are described in Table 10.

Modification of one of the prepared activated carbons, PSW-P-500, was carried out, to make a composite adsorbent, C-HFOCa-1, to be selective towards Cr(VI), As(III), As(V) and Se(IV), Se(VI) species. The modification process consists of loading and dispersing Fe(III) onto the pores of carbon in an acidic condition followed by entrapping the Fe(III) ion as Fe(OH)_3_ precipitate with Ca(OH)_2_, then washed and treated thermally to convert into hydrated iron oxide (HFeO).

### 3.3. Adsorption Studies

#### 3.3.1. Metal Ion Solutions and Reagents

Stock solutions of concentration of 1000 mg·L^−1^ of each of the Ni(II), Pb(II) and Cr(VI) species were prepared by dissolving appropriate amount of each salt, nickel (II) nitrate hexahydrate, lead (II) nitrate and potassium dichromate respectively, using deionized distilled water (DDW). Various concentrations of test solutions were prepared by subsequent dilution of the respective stock solution. The initial pH of the test solution was adjusted to the selected values using HNO_3_ and NaOH. Standard solutions of Ni(II) and Pb(II) were prepared from the dilution of the respective standard solutions (1000 mg·L^−1^) using DDW acidified earlier with 0.2% nitric acid. Standard solution of Cr(VI) was prepared from the dried salt of potassium dichromate. 1,5-Diphenylcarbazide (DPC) solution was prepared, prior to the analysis of Cr(VI) by Perkin Elmer Lambda 25 Ultra violet-visible (UV-Visible) spectrophotometer, by dissolving 0.25 g DPC in 50 mL acetone.

#### 3.3.2. Adsorption Equilibria and Kinetics

Langmuir and Freundlich adsorption isotherms [17] are widely employed to evaluate and compare the adsorption performance of adsorbents. The performances of three prepared and one commercial granular activated carbon were evaluated and compared for the removal of Ni(II) and Pb(II) in aqueous solution at an initial pH 5. The adsorption of Cr(VI) was carried out at varying pH by various adsorbents. Then, the performance of the best selected adsorbent was compared with commercial ones by fitting the adsorption equilibrium data to the Langmuir and Freundlich models.

The following Langmuir equation was used to evaluate the adsorption behavior by fitting the data.

$$q_{e}=\frac{q_{\max}bC_{e}}{1+bC_{e}}$$ (3)

where, *q*_e_ = amount of metal ion adsorbed at equilibrium per unit mass activated carbon (mg·g^−1^); *C*_e_ = equilibrium concentration of metal ion in solution (mg·L^−1^); *q*_max_ = the maximum monolayer adsorption capacity (mg·g^−1^); *b* = affinity or adsorption constant, related to the heat of adsorption, (dm^3^·g^−1^). The linear form of Equation (3) was derived as Equation (4) to determine the Langmuir parameters. Plotting *C*_e_/*q*_e_ against *C*_e_ gives a straight line with a slope 1/*q*_max_ and an intercept 1/*bq*_max_.

$$\frac{C_{e}}{q_{e}}=\frac{1}{q_{\max}}C_{e}+\frac{1}{bq_{\max}}$$ (4)

The Freundlich equation is expressed as:

qe=KFCe1n (5)

where, *q*_e_ and *C*_e_ have the same meanings as in Equation (3), *K*_F_ and “*n*” are the Freundlich empirical constants revealing the characteristic of adsorbent related to adsorption capacity and intensity respectively. The Freundlich constant, *K*_F_ unlike Langmuir constant, *q*_max_ does not predict the saturation of the solid surface by the monolayer coverage of the adsorbate [18] but it gives a relative measure in adsorption capacity and estimates bond strength [19]. The value of “*n*” discloses the adsorption pattern. The favorable adsorption is understood from the values of 1 < *n* < 10 while irreversible adsorption is noticed from *n* > 10 and unfavorable adsorption from *n* < 1.

The simplified linear logarithm form of Equation (5) is presented in Equation (6). Plotting log *q*_e_ against log *C*_e_ gives a straight line with a slope 1/*n* and an intercept log *K*_F_.

$$\text{log}q_{e}=\text{log}K_{F}+\frac{1}{n}\text{log}C_{e}$$ (6)

In batch adsorption experiment, the concentration of solute and the amount of solvent are used usually in large excess while the adsorbent is in insufficient amount. If the adsorption proceeds up to the maximum monolayer coverage and reaches the equilibrium, then we can write:

$$\text{Rate of adsorption},\;r_{\text{ad}}=\frac{dq_{t}}{dt}=k_{1\text{ad}}(q_{e}-q_{t})$$ (7)

where, *q_t_*, is the amount of adsorbate, mg·g^−1^, at any time *t*, and is equivalent to the fraction of coverage on adsorbent, θ, and *q*_e_ is the same at equilibrium and is equivalent to the unit coverage. Here, the order of adsorption is approximated to 1 with respect to (*q*_e_ − *q_t_*) and so it is pseudo first-order. If the adsorption is second-order with respect to (*q*_e_ − *q_t_*), then the Equation (7) can be written as:

$$\frac{dq_{t}}{dt}=k_{2\text{ad}}{\left(q_{e}-q_{t}\right)}^{2}$$ (8)

The constants *k*_1ad_ and *k*_2ad_ are the adsorption rate constants for the pseudo first- and pseudo second-order adsorption respectively. The differential rate law equations, Equations (7) and (8), can be solved to the integrated rate law equations, Equations (9) and (10), to determine the adsorption capacity as a function of time.

$$\text{log}\left(q_{e}-q_{t}\right)=\text{log}q_{e}-\frac{k_{1\text{ad}}}{2.303}$$ (9)

$$\frac{t}{q_{t}}=\frac{1}{k_{2}adq_{e}^{2}}+\frac{1}{q_{e}t}$$ (10)

Lagergren [20] determined the rate of adsorption for the first time, in 1898, using the pseudo first-order equation, Equation (9), and hence the equation is known as Lagergren pseudo first-order model. Equation (10) was reported by Ho and Mckay [21] for the adsorption of divalent cations and known as pseudo second-order model. For pseudo first-order adsorption, plotting log(*q*_e_ − *q_t_*) against *t* gives a straight with a slope, *k*_1ad_/2.303 and intercept, log *q*_e_ whereas for pseudo second-order, plotting *t*/*q_t_* against *t* gives a straight line with a slope, 1/*q*_e_ and an intercept, $1/k_{2\text{ad}}q_{e}^{2}$

All batch adsorption experiments were carried out at room temperature using 0.1 g dried adsorbent of various activated carbon added to 50 mL metal ion solution in a polypropylene centrifuge tube. The adsorbent concentration, however, was kept constant at 2 g·L^−1^. The adsorption equilibrium data were obtained by varying initial metal ion concentrations while the mass of activated carbon, contact time, shaking rate and initial pH were kept constant. For kinetic studies, the adsorption was carried out as a function of time keeping the initial solution concentration and pH constant. Shaking was applied placing the tubes in an orbital shaker at a rate of 160 revolutions per minute (rpm) and the equilibrium was attained in 3 days. Thereafter, the solutions were decanted and the initial and final pH of the solutions were measured using a Cyberscan-500 pH meter. The decanted Ni(II) and Pb(II) solutions were diluted using acidified (0.2% HNO_3_) DDW prior to analysis using a Perkin Elmer Analyst 400 flame atomic absorption spectrometry (AAS). The decanted Cr(VI) solutions were diluted using H_2_SO_4_ to obtain the solutions’ pH at 1 after proper dilution followed by the addition of appropriate amount of 1,5-diphenylcarbazide solution (0.5 mL DPC solution per 25 mL diluted Cr(VI) solution). After the development of color which occurred within 10 min, the solution was analyzed using UV-Visible spectrophotometer.

The following equation was used to calculate the metal uptake in mg per unit mass of adsorbent:

$$q=\frac{(C_{0}-C_{t})V}{1000m}$$ (11)

where, *q* = metal uptake mgg^−1^ adsorbent; *C*_0_ = initial concentration, mg·L^−1^; *C_t_* = concentration at any time (*t*), mg·L^−1^; *V* = volume of solution in a batch, mL; and *m* = mass of adsorbent used in a batch, g.

## 4. Conclusions

The prepared activated carbons have significantly higher adsorption capacity in removing heavy metal cations such as Ni(II) (19.6 mgg^−1^) and Pb(II) (74.6 mg·g^−1^). The commercial activated carbon has very high adsorption capacity in removing oxyanions of Cr(VI) (*q*_max_ = 71 mg·g^−1^) compared to that of prepared activated carbon (*q*_max_ = 46.30 mg·g^−1^). However, the commercial activated carbon has some limitations because of its lower adsorption capacity in the low concentration range. Hence, it cannot be suitable for drinking water purification. In contrast, acid activated carbon has the potential to scavenge some heavy metal cations and anions completely in low concentration which indicates its stronger affinity towards all heavy metal ions. Phosphoric acid activation forming surface acidic groups thus produces activated carbon suitable for removing heavy metal ions. It was observed that while commercial activated carbon has very poor affinity towards divalent cations of heavy metals, acid activated carbon has the potential to remove these cations even from very dilute solution. Although commercial activated carbon has very high adsorption capacity of Cr(VI), acid activated carbon performed better in the low concentration range. Thus, it is speculated that acid activated carbon derived from oil palm and coconut shells might be suitable to be used in drinking water purification purposes in removing heavy metal ions.

## Figures and Tables

**Table 1.** Base neutralization capacity and quantification of acidic groups on surfaces of activated carbons by Boehm’s titration method.

Sample	Base Uptake (meq·g^−1^)			
	NaHCO_3_	Na_2_CO_3_	NaOH	NaOEt
PSW-P-500	1.0	3.0	4.5	4.5
PSW-P-ad-500	1.0	3.0	4.5	4.8
CAC	–	–	0.5	–

**Sample**	**Acidic Group Quantification (meq·g^−1^)**			
	**Group I** a	**Group II** b	**Group III** c	**Group IV** d
PSW-P-500	1.0	2.0	1.5	–
PSW-P-ad-500	1.0	2.0	1.5	0.3
CAC	–	–	0.5	–

a Group I = strong acidic groups such as HO-PO_2_H_2_, OH-PO_3_H_2_, OH-PR_3_, -COOH

b Group II = phosphorous acid of intermediate strength, lactones *etc.*

c Group III = phenols, Phosphonic acids

d Group IV = carbonyl groups and NaOEt = NaOC_2_H_5_.

**Table 2.** The pH_ZPC_ of activated carbons obtained from crossover point using pH drift method.

Sl. No.	Activated Carbons	pH_ZPC_
1	PSW-P-ad-500	3.1
2	PSW-P-500	3.3
3	CAC	6.4

**Table 3.** Langmuir and Freundlich parameters for the adsorption of Ni(II) and Pb(II) at initial pH 5.

Adsorbents	Metal Ions	Langmuir Parameters				Freundlich Parameters		

		*q*_max_ (mg·g^−1^)	*B* (L·mg^−1^)	*K*_L_ (L·g^−1^)	*r*^2^	*K*_F_	*n*	*r*^2^
PSW-P-ad-500	Ni(II)	19.61 (0.334)	0.149	2.92	0.996	9.28	6.88	0.937
CPW-P-500		12.18 (0.208)	0.422	5.14	0.999	9.59	21.98	0.835
PSW-P-500		10.83 (0.185)	0.287	3.11	0.996	5.68	8.00	0.936
CAC		3.18 (0.054)	0.131	0.42	0.970	0.64	3.06	0.864

PSW-P-ad-500	Pb(II)	63.69 (0.307)	0.07	4.44	0.989	22.69	5.73	0.937
CPW-P-500		73.53 (0.355)	0.05	3.92	0.997	18.19	4.20	0.943
PSW-P-500		74.63 (0.360)	0.09	6.46	0.998	19.74	4.17	0.929
CAC		23.42 (0.113)	0.02	0.46	0.977	6.62	5.49	0.775

**Table 4.** The initial and equilibrium concentrations, and fractions of removal of divalent cations of Ni and Pb in the low concentration range at pH 5. ND = Not detectable/(ignorable amount).

Adsorbents	Metal Ions	Initial Concentration *C*_0_ (mg·L^−1^)	Equilibrium Concentration *C*_e_ (mg·L^−1^)	Removal %
CAC	Ni(II)	6.06 ± 0.05	4.14 ± 0.02	31.7 ± 0.5
		30.6 ± 0.3	27.21 ± 0.21	11 ± 1
PSW-P-500		6.06 ± 0.05	ND	100
		30.6 ± 0.3	15.05 ± 0.11	50.9 ± 0.4
CPW-P-500		6.06 ± 0.05	ND	100
		30.6 ± 0.3	9.76 ± 0.02	68.2 ± 0.2
PSW-P-ad-500		6.06 ± 0.05	ND	100
		30.6 ± 0.3	6.71 ± 0.11	78.1 ± 0.6

CAC	Pb(II)	48.7 ± 0.2	22.4 ± 0.9	53 ± 2
		71.6 ± 0.6	46 ± 1	34 ± 2
PSW-P-500		48.7 ± 0.2	3.8 ± 0.9	92 ± 2
		71.6 ± 0.6	6 ± 1	92 ± 1
CPW-P-500		48.7 ± 0.2	4.7 ± 1.2	90 ± 2
		71.6 ± 0.6	6.5 ± 1.6	91 ± 2
PSW-P-ad-500		48.7 ± 0.2	2.2 ± 0.7	96 ± 2
		71.6 ± 0.6	6.5 ± 0.2	91 ± 0.3

**Table 5.** Adsorption capacity of multiple components (Pb, Ni and Cr) at different pH values (PSW-P-ad-500 carbon dose of 1.0 g·L^−1^).

Components	Removal (%)							
	pH = 3	pH = 4	pH = 5	pH = 6	pH = 7	pH = 8	pH = 9	pH =10
*C*_0_(Pb) = 10 ppm	16	40	70	80	86	100	100	97
*C*_0_(Ni) = 3 ppm	10	36	58	80	82	95	97	95
*C*_0_(Cr) = 5 ppm	5	5	25	55	70	78	65	64

**Table 6.** Langmuir and Freundlich model parameters for the adsorption of Cr(VI).

Adsorbents	Langmuir Parameters				Freundlich Parameters		

	*q*_max_ (mg·g^−1^ )	*b* (L mg^−1^)	*K*_L_ (L·g^−1^)	*r*^2^	K_F_	*n*	*r*^2^
PSW-P-ad-500	46.30 (0.89)	0.334	15.46	0.990	17.01	3.22	0.996
CAC	70.92 (1.36)	0.092	6.52	0.984	28.57	8.91	0.807

**Table 7.** The initial and equilibrium concentrations and fractions of removal of Cr(VI) by activated carbons in the low concentration at pH 3. ND = Not detectable/(ignorable amount).

Adsorbents	Initial Concentration *C*_0_ (mg·L^−1^)	Equilibrium Concentration *C*_e_ (mg·L^−1^)	Removal (%)
CAC	39.9 ± 0.2	2.5 ± 0.3	93.7 ± 0.8
	59.4 ± 0.2	4.36 ± 0.09	92.6 ± 0.2
PSW-P-ad-500	39.9 ± 0.2	ND	100
	59.4 ± 0.2	0.7 ± 0.0	98.9 ± 0.0

**Table 8.** The rate constant, calculated adsorption equilibrium capacity and correlation coefficient of various metal ions for the adsorption onto activated carbon, PSW-P-ad-500.

Metal Ions	*k*_2ad_ (g·mg^−1^·h^−1^)	*q*_e_ (mg·g^−1^)	*r*^2^
Ni(II)	0.052	15.97	0.998
Pb(II)	0.090	39.5	1.000
Cr(VI)	0.010	33.78	0.997

**Table 9.** Preparation variables of activated carbons obtained from oil palm shell using H_3_PO_4_ in a muffle furnace.

Product Code	Impregnation		Activation	

	Soaking Time (h)	Charge State	Ramp (°C/min)	Temperature (°C)
PSW-P-500	02	wet	20	500
PSW-P-ad-500	02	semi-dried	20	500

**Table 10.** Preparation variables of activated carbons carbonized in a muffle furnace.

Products Code	Raw Materials	Pre-Treatment	Impregnation Condition	
			Charge State	Contact Time (h)
CPW-P-500	Coconut shell	30% H_3_PO_4_	Wet	02
PSW-P-500	Palm shell	30% H_2_SO_4_	Wet	02
PSW-P-ad-500	Palm shell	30% H_2_SO_4_	Semi-dried	02
